# Promoting prompt help-seeking for symptoms – assessing the impact of a gynaecological cancer leaflet on presentations to primary care: a record-based randomised control trial

**DOI:** 10.1186/s12889-018-5920-9

**Published:** 2018-08-09

**Authors:** Jackie Campbell, Kirty Vaghela, Stephen Rogers, Michelle Pyer, Alice Simon, Jo Waller

**Affiliations:** 1 Faculty of Health and Society, University of Northampton, Northampton, NN2 7AL UK; 2 Sandy Close, Wellingborough, NN8 5AY UK; 3 Department of Health Sciences, University of Leicester, 22-28 Princess Road West, Leicester, LE1 6TP UK; 4 Faculty of Health and Society, University of Northampton, Northampton, NN2 7AL UK; 5 Centre for Implementation Science, Institute of Psychiatry, Psychology and Neuroscience, King’s College London, 16 De Crespigny Park, Camberwell, London, SE5 8AF UK; 6 Department of Behavioural Science and Health, University College London, 1-19 Torrington Place, London, WC1E 6BT UK

**Keywords:** Gynaecological, Cancer, Public health, General practice, Patient education

## Abstract

**Background:**

Information leaflets have been shown to significantly improve awareness of the symptoms of gynaecological cancers and to reduce perceived barriers to seeking medical help. This record-based, parallel, randomised control trial study aimed to assess whether receipt of a leaflet would change the behaviour of women experiencing symptoms indicative of gynaecological cancers by prompting them to visit their general practitioner (GP).

**Methods:**

15,538 women aged 40 years or over registered with five general practices in Northamptonshire, UK were randomised to two groups using the SystmOne randomise facility. Those in the intervention group received an educational leaflet from their general practice explaining the symptoms of gynaecological cancers and advising symptomatic women to visit their GP. The control group were not contacted. Electronic records were interrogated to extract sociodemographic data and details of GP consultations for symptoms, tests, referrals and diagnoses relating to gynaecological cancers in the 4-month period following the mail-out of the leaflets.

**Results:**

7739 records were extracted from the intervention group and 7799 from the control group. 231 (3.0%) of the women in the intervention group, and 207 (2.7%) of the controls, presented to their GP with a relevant symptom during the 4-month period following leaflet distribution. The slightly higher rate in the intervention group did not reach statistical significance at the 5% level (RR = 1.11; 95% CI 0.92–1.33; z = 1.08; *p* = 0.28). There was a significantly lower mean time to first presentation in the symptomatic intervention group (57.2 days, sd = 36.5) compared to the control group (65.2 days, sd = 35.0) (*t* = − 2.415; *p* = 0.016). Survival analysis did not reveal a difference between the patterns of presentation in the two cohorts (Log Rank (Mantel-Cox) χ^2^ = 1.42; *p* = 0.23).

**Conclusion:**

There was no difference between intervention and control groups in the proportion of women presenting with symptoms identified in the leaflet in the four months following leaflet distribution, although the women who had been sent a leaflet presented earlier than those in the control group. A larger study is needed to test for a modest effect of leaflet distribution.

**Trial registration:**

Listed on the ISRCTN registry with study ID ISRCTN61738692 on 23–8-2017 (retrospectively registered).

## Background

Gynaecological cancers (carcinomas of the ovary, cervix, uterus, vagina, vulva or endometrium) have a combined UK incidence second only to breast cancer [1]. Earlier diagnosis of gynaecological cancers is a key strategy in closing the survival gap between England and the European average [2]. Studies suggest that a main cause of the delay in diagnosis is deferral in presentation [3–5] which is thought to be driven in part by low symptom awareness [6, 7]. Symptoms are often attributed to benign causes [8], and medical help is not sought. The GP-patient relationship is also influential in decision-making: almost half the respondents to a survey using the Cancer Awareness Measure expressed worry about ‘wasting the doctor’s time’, causing delays [9].

Leaflets can increase cancer awareness and knowledge [10–12]. However, few studies have examined whether increased awareness translates into changes in patient behaviour. There is evidence to suggest that information leaflets can increase attendance at screening [13] and reduce stroke delay [14]. Research here remains limited; no empirical research confirms that leaflets can promote symptom presentation to primary care clinicians.

An information leaflet detailing the symptoms of gynaecological cancers and encouraging women to present to their general practitioner with any concerns was developed using focus groups with experts, cancer survivors and the public [15]. It was tested with the public and shown to have at least a short-term impact; exposure to the leaflet reduced women’s perceived barriers to help-seeking, increased symptom knowledge and reduced anticipated time to help-seeking [12].

This study aimed to investigate whether being sent this leaflet affects the rate and timing of presentation to primary care for symptoms indicative of gynaecological cancers.

## Methods

### Participants and setting

The research was conducted in five general practices across Northamptonshire, UK, chosen to include a range of socio-geographical factors and practice sizes. All practices used the SystmOne electronic patient record system. Registered female patients were eligible for inclusion if they were 40 years or over (the age group in whom the majority of gynaecological cancers occur) and were not on the oncology and palliative care, learning difficulties or mental health registers.

### Sampling

Participants for the control and intervention (leaflet) groups were selected at random, without replacement, using the random selection facility within SystmOne from those women meeting the selection criteria. Approximately equal proportions of women were selected from each practice population, with equal numbers of control and intervention group participants. Those in each group were flagged using project-specific Read codes.

### Intervention

The leaflet was developed by Morris et al. (2016) [12] through an iterative process involving experts, gynaecological cancer survivors and the public. It introduces the gynaecological cancers, listing their symptoms, together with a check-list for women to record any symptoms they have had. It concludes with a ‘call to action’ for women to make a GP appointment if they have any of the symptoms. The leaflet can be found as a supplementary file to Morris et al. (2016) [12].

The leaflet was mailed to the home address of each patient selected for the intervention arm in June 2014, along with a covering letter from their GP which introduced the research project and included the sentence “I hope it [*the leaflet*] will inform you about an important health message: gynaecological symptoms should be taken seriously at every age, and discussed with your doctor if they don’t go away”.

### Outcomes

The primary outcome is the period prevalence rate for women over 40 presenting with relevant signs and symptoms within four months following distribution. We estimated the time to presentation up to four months following leaflet distribution for women seen with relevant signs and symptoms. We also calculated the proportion of women over 40 who were subsequently referred urgently for specialist assessment together with the time to urgent referral and noted gynaecological cancer diagnoses recorded during the study period, for descriptive analysis only.

### Sample size

Previous research from surveys completed using Computer Assisted Personal Interviewing (CAPI) [16] indicates that 44% of women in the general population had symptoms indicative of gynaecological symptoms in the previous 3 months. Of these, 30% had seen their GP about these symptoms. For a power of 95% of detecting a 13% increase in the attendance rate of symptomatic women seeing their GP (from 30 to 34%), 3532 symptomatic women are required in each group (α = 0.05). This effect size was considered to be clinically significant. As symptomatic women can be assumed to be 44% of the total sample group size, this required 8027 women to be selected for each of the two groups.

### Data extraction

A SystmOne query was constructed using a list of over 200 Read codes which included all relevant codes relating to the symptoms in the leaflet (130 codes), diagnostic tests ordered, test results and referrals relating to gynaecological symptoms and possible gynaecological cancer diagnoses, together with demographic information.

Patients were identified by a unique, non-identifiable project number before data was extracted for a four month period, starting the day after the mail-out date for the leaflet for each practice. Read codes were grouped under higher order categories reflecting either the leaflet content (e.g. in relation to groups of symptoms) or meaningful clinical groupings that allow for variation in codes used between different GPs.

Ethnicity recording in general practice is known to be problematic [17]. 44.6% (6926) of patients did not have their ethnicity recorded and, where it was, 91 different Read codes were used from 4 different code hierarchies. Only 4.8% of ethnicity records used the recommended 9S codes which map onto principal census categories. Comparison of ethnicities was therefore not attempted.

The GeoConvert utility [18] was used to map participant postcode districts against the Lower Layer Super Output Areas (LSOAs) within that district, together with the proportion of the district formed by each LSOA. This information was then used together with the Index of Multiple Deprivation (IMD) data for each LSOA [19] to calculate the weighted mean IMD score for each district, as a marker of area-level deprivation.

### Statistical analysis

Age and IMD score were examined using descriptive statistics. The sample characteristics of the control and intervention arm were compared using Mann-Whitney tests.

For the primary outcome, a positive outcome was defined as at least one eligible symptom Read code event recorded for the patient within the four-month study period. Cochrane’s z-tests were used to investigate differences in the proportions of women with positive outcomes in the two groups. Cochrane’s z-test was also used for comparison of urgent referral rates.

Independent t-tests were used (following confirmation of normality) for comparison of time to presentation and referral (there is no censored data for these outcomes).

Comparison of time from date of leaflet distribution to symptom presentation was subsequently investigated using Kaplan-Meier survival analysis. The start time for the event (recorded symptom) for both groups was the leaflet distribution date for the intervention group. This start date was therefore treated as a random date for the control group. This approach assumes that the presentation rate (per 4 month period) for the control group would be independent of when that time period began; the analysis investigates whether there is a difference in the distribution of presentations between the two groups. The number of days for those not having a recorded symptom by the record extraction date was censored at 4 months (123 days).

All statistical analyses were performed using two-tailed tests with statistical significance taken at the 5% level.

## Results

**Fig. 1:**
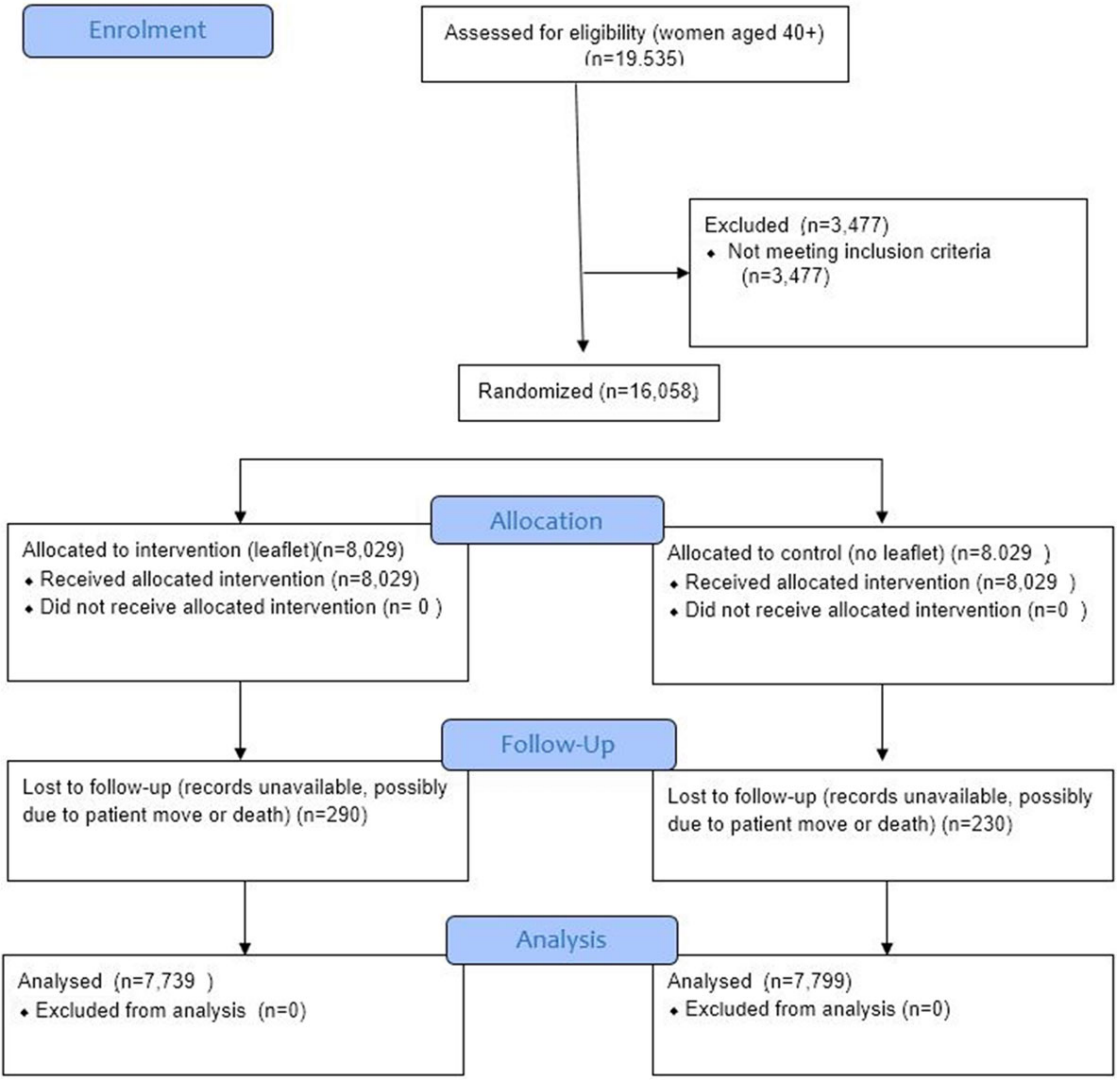
CONSORT diagram of recruitment, randomisation and analysis of intervention and control groups

7739 records were extracted from the 8029 originally identified as the intervention group and 7799 were extracted from the 8029 flagged as controls. This discrepancy is likely to be due to records becoming unavailable (e.g. because of moving from the practice or death) in the time between initial group identification and data extraction. The CONSORT flow diagram is shown in Fig. 1.

The data is available from the Zenodo repository [20].

### Participant characteristics

There was no difference between groups as assessed by age or IMD at baseline (see Table 1).

**Table 1:** Participant’s baseline characteristics

Characteristics^a^	Intervention	Control	Difference
Median age yrs. (IQR^b^)	58 (49–69)	58 (49–68)	z = 0.302, *p* = 0.76
Median IMD (IQR)	15.4 (11.96–22.98)	15.4 (11.96–22.98)	z = − 0.444, *p* = 0.66

^a^Less than 5% of records used the recommended codes for ethnicity and therefore no statistical comparison of ethnicity was performed

^b^Inter-Quartile Range

### Outcomes data

Of the 7739 women in the leaflet (intervention) group, 231 had relevant symptoms recorded by their GP in the four-month period following the leaflet distribution (3.0%). For the 7799 women in the control group, 207 had symptoms recorded in that period (2.7%). Although there is a slightly higher rate in the intervention group, this does not reach statistical significance at the 5% level (RR = 1.11; 95% CI 0.92–1.33; z = 1.08; *p* = 0.28).

The number of days from the date of the leaflet distribution to the date of the first presentation for a symptom indicative of a gynaecological cancer was calculated to investigate whether receipt of the leaflet may have an effect on the delay in symptom reporting by women. For those presenting with a relevant symptom, the mean time to presentation (from the leaflet distribution date) for the control group was 65.2 days (sd = 35.0 days). The intervention group mean time to presentation was nearly 8 days shorter (57.2 days; sd = 36.5 days). This difference was statistically significant (*t* = − 2.415, df = 474, *p* = 0.016). These results are shown in Table 2.

**Table 2:** Trial outcomes

Primary outcomes	Intervention	Control	Difference (control-intervention)
Proportion presenting	231/7799 (3.0%)	207/7739 (2.7%)	RR = 1.11, 95%CI 0.92–1.33; *p* = 0.28
Time to presentation, Mean, 95% CI	57.2 (53.4–62.0)	65.2 (60.3–70.1)	8 days; *t* = −2.42, p = 0.02
Secondary outcomes			
Proportion referred	66/7799 (0.8%)	51/7739 (0.7%)	RR = 1.28, 95%CI 0.89–1.85; p = 0.18
Time to referral Mean, 95% CI	57.1 (48.0–66.1)	67.7 (57.4–78.0)	10.6 days; *t* = −1.53, *p* = 0.13
Other			
Cancers diagnosed^a^	2	1	

^a^The numbers of cancers diagnosed was not compared statistically due to the small numbers

**Fig. 2:**
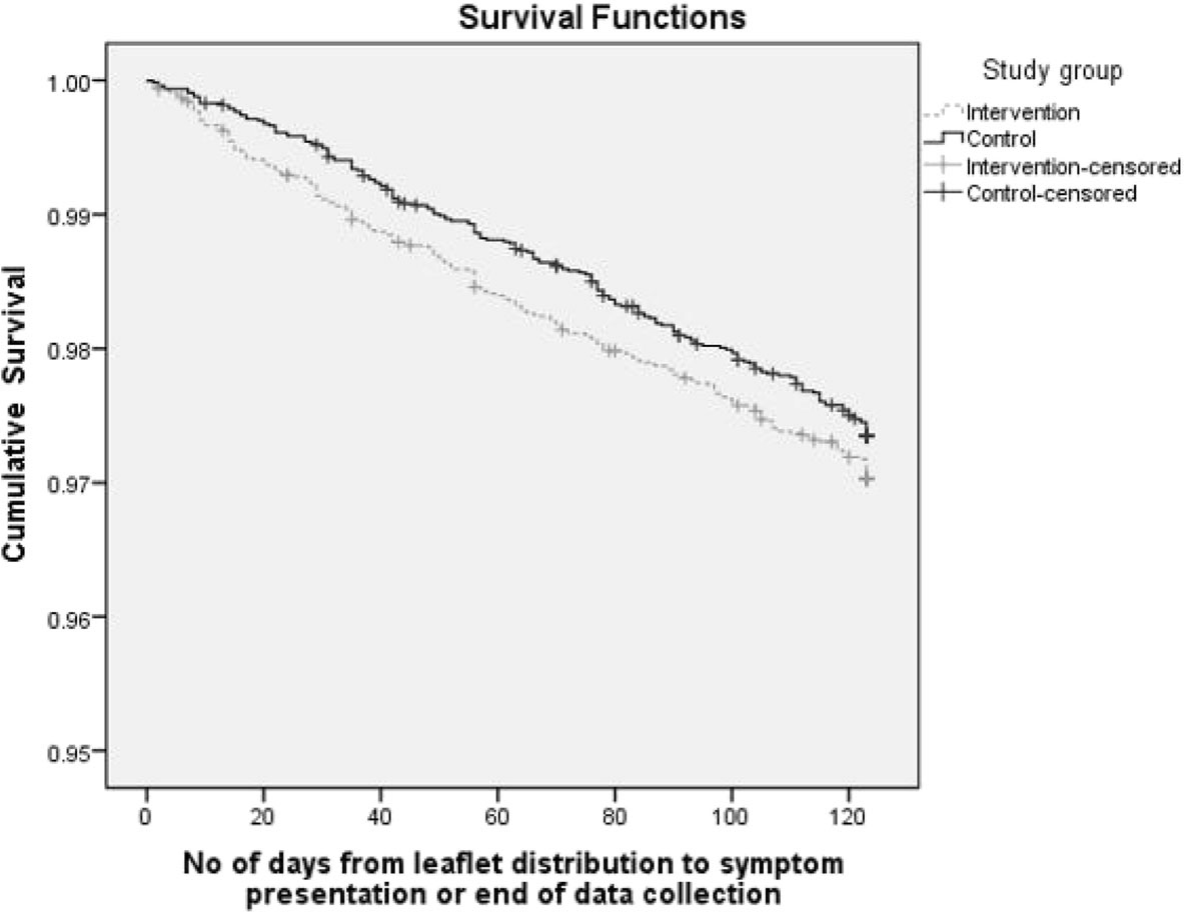
Survival functions for time to symptom presentation

Figure 2 shows the survival functions for the women in the intervention and control arms.

There was an approximately constant presentation rate over the 4-month period for the control group, whereas the intervention group showed a higher rate of presentations in the 20 days following leaflet distribution, after which the rate was approximately the same as for the control group. This difference did not reach statistical significance (Log Rank (Mantel-Cox) χ^2^ = 1.42, *p* = 0.23).

In the intervention group, 0.8% of the women (*n* = 66) were urgently referred compared to 0.7% of the women in the control group (*n* = 51). This difference did not reach statistical significance at the 5% level (RR = 1.28; 95% CI 0.89–1.85; z = 1.35; *p* = 0.18).

The number of days from leaflet distribution to the date of any urgent referral was calculated and compared between the intervention and control groups. The means were 67.7 days (sd = 36.9 days) for the control group and 57.1 days (sd = 37.1 days) for the intervention group. Although this showed a 10-day reduction in the mean time to urgent referral for the intervention group, it did not reach statistical significance (*t* = − 1.53, df = 114, *p* = 0.13).

Three gynaecological cancers were recorded as being diagnosed within the study period (four months following leaflet distribution): two in intervention group patients, and one in the control group.

## Discussion

### Summary

This study is one of the first of its kind to use a randomised controlled trial to quantify the impact of an educational leaflet on rates of, and time to, presentation for possible cancer symptoms in primary care. The differences in rate of gynaecological symptom presentation between intervention and control group were in the direction expected, but not statistically significant (3.0% compared with 2.7% (z = 1.1; *p* = 0.28)). Average time from receiving the leaflet to presentation for those with relevant symptoms was 8 days earlier in the cohort receiving the leaflet than in the control group (where the start time was the date of leaflet distribution to the comparable intervention group) (57 days compared with 65 (*t* = − 2.4, *p* = 0.02)). It was not possible to confirm a difference in survival functions when both were compared across the 4 -week follow up (Log Rank (Mantel-Cox) χ2 = 1.42, *p* = 0.23).

### Strengths and limitations

This research demonstrates the potential of record-based methods for evaluating outcomes of general practice interventions. The methodology enabled the testing of a simple intervention across five practices with modest resources. Sources of bias that can affect observational studies were excluded by randomising the distribution of leaflets. Age and index of multiple deprivation were compared between the cohorts, confirming broad similarities. Primary and secondary outcomes were defined in advance with sample size drawn from a separate study that aimed to estimate the likely symptom frequency in general practice populations [16]. Data was extracted and compiled without reference to allocation to intervention or control groups.

As with all record-based studies, the coded items available and extracted may not identify all relevant patients, although the effect of this is likely to be similar in intervention and control groups. In this study, the rate of symptom presentation based on electronic records was considerably less than predicted from estimates based on a questionnaire study [16] which had been used for the a priori sample size calculation. This may have been due to inflated symptom incidence due to survey self-selection bias: it is possible that the questions relating to gynaecological cancer symptoms may have been differentially answered by those having symptoms. Conversely, there may be an under-reporting of symptoms by GPs on electronic patient records. Although the relative risk observed was similar to that used in the sample size calculation (observed RR = 1.11 compared to estimated RR = 1.13), the observed incidence was approximately one-tenth of the previously published estimate. The actual power of the study was therefore only 20% (α = 0.05) and our study cannot exclude an effect of leaflet distribution on help seeking that could be of importance.

### Comparison with existing literature

Few trials of interventions directed towards early presentation of patients with cancer-related symptoms exist, though it is known that targeted public information leaflets can increase awareness of cancer symptoms [10–12]. A bowel cancer screening study in which leaflets sent to individual patients from practices increased participation in bowel cancer screening programmes by 3–4% [13] and a study investigating the impact of leaflets on presentation of stroke symptoms delivered as part of a multifaceted campaign [14] are particularly relevant. However, other trials of leaflets promoting colorectal cancer screening have been less successful [21].

This study is one of the first to attempt to systematically quantify the impact of an educational leaflet on rates of, and time to, presentation for potential gynaecological cancer symptoms in primary care. A prior evaluation of the leaflet used in this study demonstrated that its impact on patients’ anxiety levels was negligible and changes in anxiety did not influence patients’ intentions to seek help [12]. Some evidence for increased rates of presentation in the three weeks after receiving the leaflet was found, but this was a non-significant trend. There was, however, a difference in time to presentation, with women in the intervention group presenting with symptoms just over a week faster than those in the control group. Evidence which quantifies the specific effect of delay to presentation to clinical outcome is lacking in the current literature and the evidence for diagnostic delay is confounded by large effects due to the type of cancer and stage of diagnosis, amongst other factors [2, 22]. However, given that the NHS has a target of 14 days from presentation at GP to specialist consultation for suspected cancers, an additional delay of 8 days is intuitively clinically important and is an important effect in the context of possible public health impact.

### Implications for research and practice

The National Awareness and Early Diagnosis Initiative (NAEDI) was launched in the United Kingdom as part of a strategy to improve cancer outcomes. The key hypothesis underpinning NAEDI was that delays lead to cancer patients being diagnosed with more advanced disease and thus experiencing poor 1-year and 5-year survival rates. An investigative phase was followed by piloting and then definitive regional, disease -focused campaigns directed towards raising public awareness of symptoms and signs of common cancers [23]. The model adopted was typically focused on single awareness-raising messages using posters and leaflet distribution in conjunction with social marketing through radio and television. Campaigns have had variable outcomes, with one campaign to promote early diagnosis of lung cancer claiming some success [24], and mixed results from campaigns directed towards bowel cancer, urological cancer and ovarian cancer [25–28].

Attempts have been made to evaluate process and outcome, with urgent cancer referrals most often used as a proxy outcome. Where effects have been noted it remains difficult to be clear what impact the components of different campaigns might have, and in particular the value of local distribution of leaflets. Evidence that a low-intensity intervention such as leaflet distribution may decrease the patient interval for potential cancer symptoms lends support to pursuing this type of approach as part of a wider strategy for reducing the time to cancer diagnosis. This study does not provide reliable estimates for the likely impact on GP consultations or urgent referrals, but these could be derived in a larger randomised trial or observational study.

## Conclusions

This study is one of the first to attempt to systematically quantify the impact of an educational leaflet on rates of, and time to presentation for potential cancer symptoms in primary care. There was some evidence for increased rates of presentation in the three weeks after receiving the leaflet, but this was not statistically significant. There was a significant difference in time to presentation, with women in the intervention group presenting with symptoms just over a week faster than those in the control group. The evidence that this low-intensity intervention may decrease time to presentation for potential cancer symptoms lends support to pursuing this type of approach as part of a wider strategy for reducing the time to cancer diagnosis.

## Abbreviations

CAPI: Computer Assisted Personal Interviewing
CI: Confidence Interval
CONSORT: Consolidated Standards of Reporting Trials
GP: General Practitioner
IMD: Index of Multiple Deprivation (IMD)
LSOA: Lower Layer Super Output Areas
n: number
NRES: National Research Ethics Service
RCT: Randomised Control Trial
REC: Research Ethics Committee
RR: Relative Risk
sd: Standard deviation
UK: United Kingdom
